# Assessing the Predictive Utility of the C-Reactive Protein-to-Lymphocyte Ratio for Mortality in Isolated Traumatic Brain Injury: A Single-Center Retrospective Analysis

**DOI:** 10.3390/diagnostics14182065

**Published:** 2024-09-18

**Authors:** Ching-Ya Huang, Shao-Chun Wu, Yuan-Hao Yen, Johnson Chia-Shen Yang, Shiun-Yuan Hsu, Ching-Hua Hsieh

**Affiliations:** 1 Department of Plastic Surgery, Kaohsiung Chang Gung Memorial Hospital, Chang Gung University College of Medicine, Kaohsiung 83301, Taiwan; b101106030@tmu.edu.tw (C.-Y.H.); bloodguardian@gmail.com (Y.-H.Y.); prs581126@gmail.com (J.C.-S.Y.); 2 Department of Anesthesiology, Kaohsiung Chang Gung Memorial Hospital, Chang Gung University College of Medicine, Kaohsiung 83301, Taiwan; shaochunwu@gmail.com; 3 Department of Trauma Surgery, Kaohsiung Chang Gung Memorial Hospital, Chang Gung University College of Medicine, Kaohsiung 83301, Taiwan; ah.lucy@hotmail.com

**Keywords:** trauma, mortality, prognosis, traumatic brain injury (TBI), C-reactive protein to lymphocyte ratio (CLR)

## Abstract

**Introduction:** Early identification of high-risk traumatic brain injury (TBI) patients is crucial for optimizing treatment strategies and improving outcomes. The C-reactive protein-to-lymphocyte ratio (CLR) reflects systemic immunology and inflammation function and serves as a new biomarker for patient stratification. This study aimed to assess the predictive value of the CLR for mortality in patients with isolated moderate to severe TBI. **Methods:** A retrospective analysis of trauma registry data from 2009 to 2022 was conducted, including 1641 adult patients with isolated moderate to severe TBI. Patient demographics, the CLR, injury characteristics, and outcomes were compared between deceased and surviving patients. Univariate and multivariate analyses were performed to identify mortality risk factors. The optimal CLR cut-off value for predicting mortality was determined using receiver operating characteristic (ROC) curve analysis. **Results:** The CLR was significantly higher in deceased patients compared to survivors (60.1 vs. 33.9, *p* < 0.001). The optimal CLR cut-off value for predicting mortality was 54.5, with a sensitivity of 0.328 and a specificity of 0.812. The area under the ROC curve was 0.566, indicating poor discriminative ability. In the multivariate analysis, the CLR was not a significant independent predictor of mortality (OR 1.03, *p* = 0.051). After propensity score matching to attenuate the difference in baseline characteristics, including sex, age, comorbidities, conscious level, and injury severity, the high-CLR group (CLR ≥ 54.5) did not have significantly higher mortality compared to the low-CLR group (CLR < 54.5). **Conclusion:** While the CLR was associated with mortality in TBI patients, it demonstrated poor discriminative ability as a standalone predictor. The association between a high CLR and worse outcomes may be primarily due to other baseline patient and injury characteristics, rather than the CLR itself.

## 1. Introduction

Traumatic brain injury (TBI) remains a leading cause of death and long-term disability globally [1]. Various injury mechanisms, severities, and patient responses influence the outcome of TBI, making accurate patient prognostication challenging in clinical settings. To predict the outcomes, such as mortality and functional recovery, some advanced multivariable prognostic models have been developed by incorporating a wider range of variables, including age, motor response, pupillary reactivity, and image findings. For instance, the International Mission for Prognosis and Analysis of Clinical Trials in Traumatic Brain Injury (IMPACT) and the Corticoid Randomization After Significant Head injury (CRASH) models have been extensively validated across large patient cohorts. The IMPACT model has shown an area under the receiver operating characteristic curve (AUC) ranging from 0.66 to 0.84 for predicting mortality, indicating strong predictive power [2]. Similarly, the CRASH model, developed from a cohort of over 10,000 patients, has demonstrated AUC values between 0.79 and 0.83 in different validation studies [3]. However, in urgent situations, acquiring comprehensive data for these scoring systems often takes time.

Early stratification of TBI patients is crucial for optimizing treatment strategies and improving outcomes, particularly in the acute phase when timely interventions can significantly influence recovery. Rapid and also accurate assessment allows clinicians to enable the appropriate allocation of resources and the implementation of tailored therapeutic approaches. Simple prognostic factors, such as Glasgow Coma Scale (GCS) scores and specific injury types, offer several advantages in this context [4]. These tools are simple to use, require minimal equipment, and are applicable in a variety of settings. However, these simple prognostic tools are often insufficient to capture the complexity of TBI outcomes. Therefore, there is an increasing awareness of the necessity to investigate alternative biomarkers for prognosis prediction.

C-reactive protein (CRP) levels in trauma patients rapidly rise soon after injury, peaking around the third day, and are correlated with the extent of trauma and tissue damage [5]. After trauma, there is a rapid increase in lymphocyte count within the first 3 h post-injury, followed by a sharp decline in the subsequent 6–72 h. This decrease in lymphocytes, known as lymphopenia, is associated with increased secondary tissue injury and a higher risk of complications such as multiple organ dysfunction syndrome and sepsis. Persistent lymphopenia beyond the acute phase correlates with poorer outcomes, including prolonged hospital stays and increased mortality [6,7]. This acute response following trauma, marked by a rise in CRP and a decrease in lymphocyte levels, plays a crucial role in the outcomes of patients [8,9,10,11]. The discovery of the C-reactive protein-to-lymphocyte ratio (CLR) as a possible biomarker opens new opportunities for improving early classification efforts in critically ill patients. The CLR, which combines these two markers, provides a comprehensive measure of the body’s response to injury, reflects systemic immunology and inflammation function, and serves as a rapid and potential prognosis indicator in a variety of clinical contexts, including SARS-CoV-2, acute pancreatitis, dilated cardiomyopathy, and oncology [12,13,14,15,16]. The CLR, being a readily available and easily interpretable biomarker for immunology and inflammatory responses after injury, may be used as a prognostic marker for TBI patients. Hence, the objective of this study is to assess the predictive significance of the CLR in isolated moderate to severe TBI patients by retrospectively analyzing trauma registry data from a level I trauma hospital.

## 2. Methods

### 2.1. Patient Enrollment and Study Design

This is a retrospective study approved by the Institutional Review Board (IRB) of Chang Gung Memorial Hospital under approval number 202400890B0. Following IRB guidelines, patient consent was not required as the study involved a review of pre-registered trauma data. Medical records from the Trauma Registry System at a trauma center in southern Taiwan, covering the period from 1 January 2009, to 31 December 2022, were analyzed. This study included all trauma patients aged 20 years or older with a head Abbreviated Injury Scale (AIS) score of ≥3. Patients with burns, hanging injuries, and those who drowned or had unavailable laboratory data were excluded. The study’s approach included detailed documentation of patients’ sex, age, comorbidities, CLR, GCS, AIS, injury severity score (ISS), in-hospital mortality, and length of stay in the hospital. The CLR is computed by dividing the patient’s CRP level (mg/dL) by their lymphocyte count (10^9^/L).

### 2.2. Statistical Analysis

The chi-square test was performed to compare the proportions of categorical variables in deceased patients versus those who survived, yielding odds ratios (OR) and 95% confidence intervals (CI). Levene’s test was performed to confirm variance homogeneity, followed by an ANOVA to examine the differences between continuous variables. The Mann–Whitney U test was used to assess non-normally distributed continuous data, and the results were provided as the median and interquartile range. Univariate and multivariate analyses were conducted to investigate the independent risk variables for death. Using the Youden index, the optimal cut-off value for the CLR’s best prediction performance was determined, and the area under the receiver operating characteristic curve (AUC of ROC) was calculated [17,18]. Statistical analyses were performed with IBM SPSS Statistics, Version 23, at a significance level of *p* < 0.05.

## 3. Results

### 3.1. Patients in the Study Cohort

**Figure 1:**
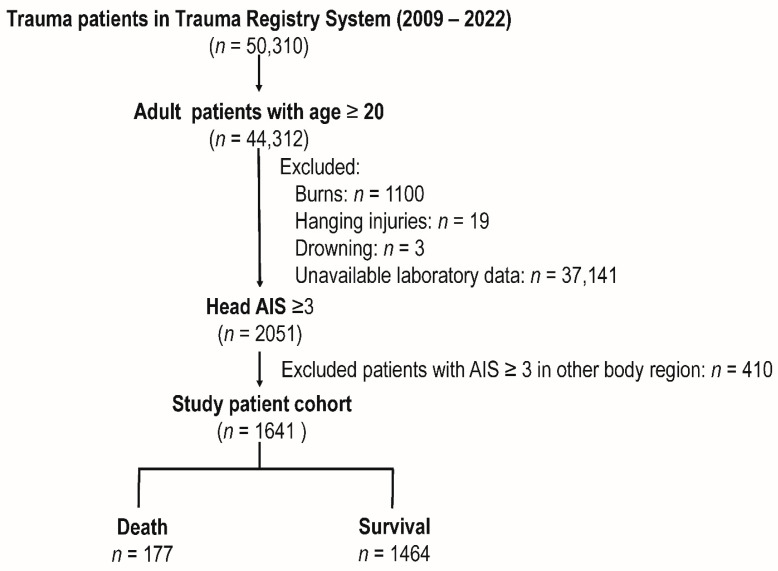
Enrollment process of the adult traumatic brain injury (TBI) patients into the study cohort. AIS, Abbreviated Injury Scale.

The study cohort included 50,310 trauma patients from the Trauma Registry System between 2009 and 2022 (Figure 1). Of these, 44,312 were adult patients aged 20 and up. We subsequently excluded patients based on specific exclusion criteria, which included burns (*n* = 1100), hanging injuries (*n* = 19), drowning (*n* = 3), and unavailable laboratory data (*n* = 37,141). This yielded 2051 individuals with a head AIS score of ≥3. After excluding individuals with AIS scores ≥ 3 (*n* = 410) in other body regions, the final research population was 1641, including 177 deaths and 1464 patients who survived.

### 3.2. Demographic and Clinical Characteristics of Patients Stratified by Outcomes

Table 1 presents the demographic and clinical characteristics of the deceased patients and those who survived within the study cohort. Deceased patients were predominantly male (70.6% vs. 64.5%) and significantly older (mean age 67.0 vs. 60.5 years, *p* < 0.001) than patients who survived. The deceased patients exhibited a significantly higher CLR (60.1 vs. 33.9, *p* < 0.001) and elevated CRP levels (66.6 vs. 41.8 mg/L, *p* < 0.001) than patients who survived. The prevalence of comorbidities such as coronary artery disease (CAD) (18.1% vs. 8.8%, *p* < 0.001) and end-stage renal disease (ESRD) (11.3% vs. 2.9%, *p* < 0.001) was significantly higher among deceased patients than patients who survived. Patients who died had lower GCS scores, a median score of 7 compared to 15 for survivors (*p* < 0.001), and worse injuries, as shown by a higher ISS (median 25 vs. 16 for survivors; *p* < 0.001) than patients who lived. In comparison with those who survived, the length of hospital stay was shorter for deceased patients (mean 11.8 vs. 16.5 days, *p* < 0.001).

### 3.3. Univariate and Multivariate Analysis of Factors Associated with Mortality

Table 2 summarizes the univariate and multivariate analyses of risk factors associated with mortality in isolated moderate to severe TBI patients. In the univariate analysis, a number of factors were significantly associated with increased mortality. Older age was associated with an increased risk of mortality (OR 1.02, *p* < 0.001). A higher CLR was also linked to an elevated risk of death (OR 1.04, *p* < 0.001), as were higher CRP levels (OR 1.06, *p* < 0.001). Among comorbidities, CAD (OR 2.28, *p* < 0.001) and ESRD (OR 4.31, *p* < 0.001) were significantly associated with mortality. Lower GCS scores (OR 0.83, *p* < 0.001) and higher ISS (OR 1.18, *p* < 0.001) were also predictors of increased mortality. In the multivariate analysis, older age (OR 1.03, *p* < 0.001), CAD (OR 2.05, *p* = 0.005), ESRD (OR 4.69, *p* < 0.001), lower GCS scores (OR 0.86, *p* < 0.001), and higher ISS (OR 1.14, *p* < 0.001) remained significant independent risk factors for mortality. CRP levels were not significant predictors of mortality after adjusting for other variables (OR 1.00, *p* = 0.944). Additionally, although higher CLR showed a trend towards increased mortality (OR 1.03, *p* = 0.051), it did not reach statistical significance in the multivariate analysis.

### 3.4. The Optimal Cut-Off Value of CLR in Predicting Mortality

**Figure 2:**
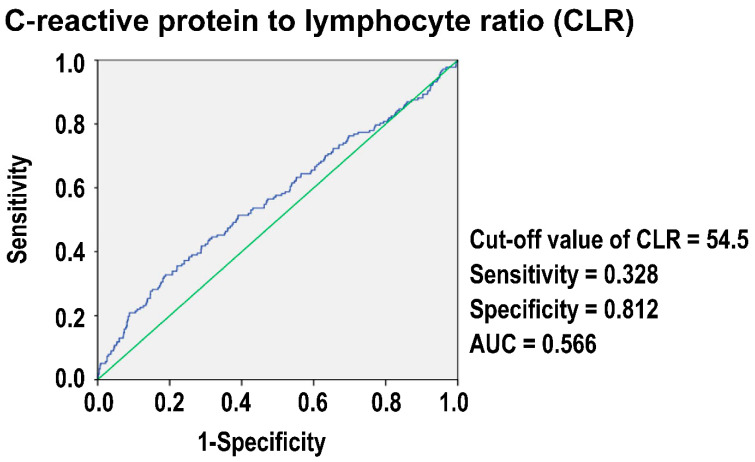
Performance characteristics of the C-reactive protein-to-lymphocyte ratio (CLR) for predicting mortality in traumatic brain injury (TBI) patients.

Figure 2 illustrates the ROC curve for the CLR in predicting mortality among isolated moderate to severe TBI patients. The analysis identified an optimal cut-off value of 54.5 for the CLR. At this threshold, the sensitivity of the CLR for predicting mortality was 0.328, and the specificity was 0.812. The AUC was 0.566, indicating a relatively low discriminative power of the CLR as a prognostic marker for mortality in this patient population.

### 3.5. Comparative Demographics and Outcomes Based on Grouping by CLR

When these patients were stratified into high (≥54.5)- and low (<54.5)-CLR groups (Table 3), patients in the high-CLR group were more likely to be male (71.3% vs. 63.7%, *p* = 0.009) and older (mean age 66.6 vs. 59.8 years, *p* < 0.001). Comorbidities such as hypertension (HTN) (50.9% vs. 38.3%, *p* < 0.001), diabetes mellitus (DM) (27.2% vs. 21.8%, *p* = 0.035), and ESRD (5.7% vs. 3.3%, *p* = 0.040) were more prevalent in the high-CLR group than those with a low CLR. The GCS scores were significantly lower in the high-CLR group, with a median score of 13 compared to 15 in the low-CLR group (*p* = 0.003). Patients with a higher CLR had a greater incidence of severe injuries, with a significantly higher proportion of patients having an ISS ≥ 25 (26.3% vs. 18.7%, *p* = 0.002). Mortality was significantly higher in the high-CLR group than the low-CLR group (17.4% vs. 9.1%, *p* < 0.001), with an OR of 2.10, indicating a more than twofold increase in the risk of death. Additionally, patients in the high-CLR group experienced significantly longer hospital stays (mean 19.0 vs. 15.2 days, *p* < 0.001) than those with a low CLR. These findings suggest that the isolated moderate to severe TBI patients with a high CLR were associated with worse clinical outcomes, including increased mortality and longer hospitalization.

### 3.6. Propensity-Score-Matched Analysis of Patients Grouped by CLR

Table 4 displays the results following propensity score matching for the CLR groups (≥54.5 vs. <54.5), after adjusting for baseline patient and injury variables. Both groups had similar sex, age, comorbidity, GCS, and ISS profiles. Among these propensity-score-matched patient cohorts, the high-CLR group had no significant difference in mortality (15.8% vs. 11.7%, *p* = 0.154) or longer hospital stays (19.5 vs. 17.4 days, *p* = 0.121) compared to the low-CLR group. These findings revealed that, after controlling for confounders, patients with a high CLR were not associated with worse outcomes than those with a low CLR, implying that the difference in mortality rates between patients with a high and low CLR may be primarily due to baseline patient and injury characteristics.

## 4. Discussion

This study assessed the predictive value of the CLR in isolated moderate to severe TBI patients and found that the overall discriminative ability of the CLR alone (AUC = 0.566) for mortality was poor, and the CLR did not appear to be a significant independent risk factor for predicting mortality in the multivariate analysis. After propensity score matching to reduce baseline patient and injury characteristic variation, patients with a high CLR did not have worse outcomes than those with a low CLR. This finding implies that, while a greater CLR may be related to worse outcomes in isolated moderate to severe TBI patients, the CLR alone was not a valid predictive marker in predicting a worse outcome.

Many studies have demonstrated the usefulness of the CLR as a prognostic tool in various illnesses. For example, in older patients following hip fracture repair, research has identified the CLR as a significant prognostic factor for predicting 30-day death [19]. The patients who died within 30 days had much greater preoperative CLR levels than survivors [19]. Tonduangu et al. [12] also found that CLR thresholds of 78.3 and 159.5 were good at predicting infection and death in COVID-19 patients, with sensitivity levels of 79% and specificities of 47% and 70%. Researchers found that a lower CLR cut-off of 21.25 for the Omicron BA.2.2 variation was predictive of worse outcomes, with 72.3% sensitivity and 86% specificity [20]. A CLR cut-off of 30.835 demonstrated good predictive ability in the setting of acute pancreatitis, with 73.7% sensitivity and 88.6% specificity [16]. Also, the CLR was very good at telling the difference between acute and perforated appendicitis, with an AUC of 0.83 and an ideal cut-off of 0.45 [21]. It had 70% sensitivity and 96% specificity. On the other hand, our research found that a CLR cut-off of 54.5 was the best way to predict death in isolated moderate to severe TBI patients, with a low sensitivity of 0.328 and a high specificity of 0.812. The reason that the CLR did not achieve significance in predicting mortality in isolated moderate to severe TBI patients may be due to the unique nature of this population. However, systemic immune and inflammation change is a more central aspect of the disease process, thereby offering a clearer prognostic value [12,13,14,15,16,21,22]. TBI patients often need highly specialized and tailored care, with the timing and success of surgical procedures, intracranial pressure control, and prevention of secondary brain injury being important predictors of outcomes [23,24,25]. The severity of neurological damage in an acute episode, as well as the effectiveness of rapid surgical procedures, frequently have a more direct impact on the outcome in TBI patients. The prognosis in TBI is mostly determined by neurological conditions and surgical results, which may be underrepresented in systemic immunology and inflammation indicators such as the CLR. For example, high intracranial pressure may accurately reflect the extent of brain damage and the likelihood of secondary brain damage [23,25]. The severity of the brain injury, as assessed by clinical instruments such as the GCS and ISS, is more important in determining patient prognosis [26,27,28,29] than the CLR itself. Furthermore, the lack of significance of the CLR in predicting mortality in TBI patients shows the population’s unique and complex characteristics. Compared to simple scores like the CLR, some well-developed multivariable models are likely to perform better in the complicated context of TBI [30].

CRP plays a significant role in the prognosis and management of patients with TBI. Elevated CRP levels are consistently associated with poor outcomes. High-sensitivity CRP levels within two weeks of TBI correlate strongly with severe disability and poor outcomes at six months [31]. Additionally, CRP measured early after trauma is particularly useful in assessing diffuse brain injuries, as higher levels are found in patients with low GCS scores and poor clinical outcomes [32]. The combination of CRP with other biomarkers, such as D-dimer, enhances its diagnostic value in detecting brain lesions on CT scans, as patients with lesions had significantly higher CRP levels (11.12 ± 1.96 mg/L) compared to those without lesions (3.94 ± 0.39 mg/L) [33]. However, in contrast to the typical association between lymphopenia and poor outcomes in trauma patients, our study found that the mean lymphocyte count was actually higher in the deceased group compared to the survivors [6,7]. This unexpected finding suggests that the elevated CLR observed in the deceased group was primarily driven by higher CRP levels rather than a decrease in lymphocyte count. While lymphopenia is commonly linked with increased secondary tissue injury and complications such as multiple organ dysfunction syndrome and sepsis [6,7], our data suggest that in this cohort of TBI patients, the role of lymphocytes may differ. This discrepancy highlights the complexity of immune responses in TBI and suggests that further research is needed to explore the relationship between lymphocyte levels and outcomes in this population. Furthermore, there are few studies that have discussed the role of the CLR in TBI patients. The CLR has been shown to be a valuable predictor in other forms of brain injury, such as aneurysmal subarachnoid hemorrhage, where elevated levels were associated with poor outcomes, including cerebral vasospasm and delayed cerebral ischemia. In aneurysmal subarachnoid hemorrhage, patients with high CLR levels upon admission were more likely to experience these complications, indicating the importance of systemic inflammation in the prognosis of brain injuries [34]. Given the similarities in the inflammatory processes in TBI, the CLR may potentially serve as a useful marker for predicting complications and guiding treatment in TBI patients, although more research is needed to validate this in TBI-specific contexts.

This study has several additional limitations that should be acknowledged. First, as a retrospective single-center study, the findings may not be generalizable to all trauma populations or healthcare settings, and the potential for selection bias cannot be ruled out. Second, the unbalanced number of survivors compared to mortalities could have influenced the results and the overall interpretation of the CLR’s predictive value. Additionally, the exclusion of patients with incomplete data, particularly those lacking laboratory results, may have introduced bias if these patients systematically differed from the included cohort. Third, some relevant clinical variables, such as detailed information on the timing of interventions or the presence of resuscitation, may not have been fully captured. Fourth, the study’s focus on in-hospital mortality as the primary outcome may overlook other important dimensions of TBI prognosis, such as functional outcomes, quality of life, or long-term survival, which are crucial for a comprehensive assessment of the CLR’s prognostic value. Additionally, the study did not account for potential changes in the CLR over time or the impact of ongoing treatment interventions, which could provide valuable insights into the dynamic nature of the inflammatory response in TBI patients.

## 5. Conclusions

In this study, the CLR demonstrated limited prognostic value for mortality in isolated moderate to severe TBI patients. Despite its association with mortality, the CLR lacked independent predictive power when adjusted for confounding factors. The observed relationship between an elevated CLR and adverse outcomes likely stems from underlying patient and injury characteristics rather than the CLR itself. Future research is encouraged to explore the potential integration of the CLR into comprehensive prediction models to enhance prognostication in TBI patients.

## Figures and Tables

**Table 1:** Patient and injury characteristics of the deceased patients and patients who survived.

Variables	Death *n* = 177	Survival *n* = 1464	OR (95%CI)	*p*
Sex				0.109
Male, *n* (%)	125(70.6)	945(64.5)	1.32(0.94–1.86)	
Female, *n* (%)	52(29.4)	519(35.5)	0.76(0.54–1.07)	
Age, years (mean ± SD)	67.0 ± 17.2	60.5 ± 19.1	-	<0.001
CLR	60.1 ± 111.5	33.9 ± 55.1	-	<0.001
CRP (mg/L)	66.6 ± 84.4	41.8 ± 52.5	-	<0.001
Lymphocyte (10^9^/L)	1.9 ± 1.4	1.8 ± 1.3	-	0.390
Comorbidities				
CVA, *n* (%)	14(7.9)	108(7.4)	1.08(0.60–1.93)	0.799
HTN, *n* (%)	84(47.5)	586(40.0)	1.35(0.99–1.85)	0.057
CAD, *n* (%)	32(18.1)	129(8.8)	2.28(1.50–3.49)	<0.001
CHF, *n* (%)	0(0.0)	11(0.8)	-	0.247
DM, *n* (%)	42(23.7)	334(22.8)	1.05(0.73–1.52)	0.784
ESRD, *n* (%)	20(11.3)	42(2.9)	4.31(2.47–7.53)	<0.001
GCS, median (IQR)	7(3–14)	15(10–15)	-	<0.001
3–8, *n* (%)	97(54.8)	292(19.9)	4.87(3.53–6.72)	<0.001
9–12, *n* (%)	21(11.9)	188(12.8)	0.91(0.57–1.48)	0.713
13–15, *n* (%)	59(33.3)	984(67.2)	0.24(0.18–0.34)	<0.001
ISS, median (IQR)	25(16–25)	16(16–20)	-	<0.001
1–15, *n* (%)	11(6.2)	327(22.3)	0.23(0.12–0.43)	<0.001
16–24, *n* (%)	57(32.2)	914(62.4)	0.29(0.21–0.40)	<0.001
≥25, *n* (%)	109(61.6)	223(15.2)	8.92(6.38–12.47)	<0.001
Hospital stay (days)	11.8 ± 17.0	16.5 ± 14.6	-	<0.001

CAD = coronary artery disease; CHF = congestive heart failure; CI = confidence interval; CVA = cerebral vascular accident; CRP = C-reactive protein; CLR = CRP (mg/L)/lymphocyte (10^9^/L); DM = diabetes mellitus; ESRD = end-stage renal disease; GCS = Glasgow Coma Scale; HTN = hypertension; IQR = interquartile range; ISS = injury severity score; OR = odds ratio; SD = standard deviation.

**Table 2:** Univariate and multivariate analysis of factors associated with mortality in traumatic brain injury (TBI) patients.

Variables	Univariate Analysis			Multivariate Analysis		
	OR	(95% CI)	*p*	OR	(95% CI)	*p*
Age (years)	1.02	(1.01–1.03)	<0.001	1.03	(1.02–1.04)	<0.001
CLR	1.04	(1.02–1.06)	<0.001	1.03	(1.00–1.07)	0.051
CRP (mg/L)	1.06	(1.04–1.08)	<0.001	1.00	(0.97–1.04)	0.944
Lymphocyte (10^9^/L)	1.05	(0.94–1.17)	0.390	1.00	(0.87–1.15)	0.984
CAD, yes	2.28	(1.50–3.49)	<0.001	2.05	(1.24–3.38)	0.005
ESRD, yes	4.31	(2.47–7.53)	<0.001	4.69	(2.37–9.27)	<0.001
GCS, yes	0.83	(0.80–0.85)	<0.001	0.86	(0.83–0.90)	<0.001
ISS	1.18	(1.14–1.22)	<0.001	1.14	(1.10–1.18)	<0.001

CAD = coronary artery disease; CI = confidence interval; CRP = C-reactive protein; CLR = CRP (mg/L)/lymphocyte (10^9^/L); ESRD = end-stage renal disease; GCS = Glasgow Coma Scale; ISS = injury severity score; OR = odds ratio.

**Table 3:** Comparative analysis of the patients with a high and low C-reactive protein-to-lymphocyte ratio (CLR) based on the optimal cut-off value of 54.5.

	CLR			
	≥54.5 *n* = 334	<54.5 *n* = 1307	OR (95%CI)	*p*
Sex				0.009
Male, *n* (%)	238(71.3)	832(63.7)	1.42(1.09–1.84)	
Female, *n* (%)	96(28.7)	475(36.3)	0.71(0.54–0.92)	
Age, years (mean ± SD)	66.6 ± 17.2	59.8 ± 19.2	-	<0.001
Comorbidities				
CVA, *n* (%)	31(9.3)	91(7.0)	1.37(0.89–2.09)	0.149
HTN, *n* (%)	170(50.9)	500(38.3)	1.67(1.31–2.13)	<0.001
CAD, *n* (%)	42(12.6)	119(9.1)	1.44(0.99–2.09)	0.057
CHF, *n* (%)	2(0.6)	9(0.7)	0.87(0.19–4.04)	0.858
DM, *n* (%)	91(27.2)	285(21.8)	1.34(1.02–1.77)	0.035
ESRD, *n* (%)	19(5.7)	43(3.3)	1.77(1.02–3.09)	0.040
GCS, median (IQR)	13(8–15)	15(9–15)	-	0.003
3–8, *n* (%)	92(27.5)	297(22.7)	1.29(0.98–1.70)	0.064
9–12, *n* (%)	55(16.5)	154(11.8)	1.48(1.06–2.06)	0.022
13–15, *n* (%)	187(56.0)	856(65.5)	0.67(0.53–0.86)	0.001
ISS, median (IQR)	16(16–25)	16(16–20)	-	0.008
1–15, *n* (%)	51(15.3)	287(22.0)	0.64(0.46–0.89)	0.007
16–24, *n* (%)	195(58.4)	776(59.4)	0.96(0.75–1.23)	0.743
≥25, *n* (%)	88(26.3)	244(18.7)	1.56(1.18–2.06)	0.002
Mortality, *n* (%)	58(17.4)	119(9.1)	2.10(1.49–2.95)	<0.001
Hospital stay (days)	19.0 ± 16.5	15.2 ± 14.5	-	<0.001

CAD = coronary artery disease; CHF = congestive heart failure; CI = confidence interval; CVA = cerebral vascular accident; CRP = C-reactive protein; CLR = CRP (mg/L)/ lymphocyte (10^9^/L); DM = diabetes mellitus; ESRD = end-stage renal disease; GCS = Glasgow Coma Scale; HTN = hypertension; IQR = interquartile range; ISS = injury severity score; OR = odds ratio; SD = standard deviation.

**Table 4:** Propensity-score-matched analysis of patients grouped by C-reactive protein-to-lymphocyte ratio (CLR).

	CLR							
	≥54.5 *n* = 298		<54.5 *n* = 298		OR (95%CI)		*p*	SD
Sex								
Male, *n* (%)	216	(72.5)	216	(72.5)	1.00	(0.70–1.43)	1.000	0.00%
Age, years	65.2	±17.3	65.0	±17.3	-		0.898	1.05%
Comorbidities								
CVA, *n* (%)	19	(6.4)	19	(6.4)	1.00	(0.52–1.93)	1.000	0.00%
HTN, *n* (%)	147	(49.3)	147	(49.3)	1.00	(0.73–1.38)	1.000	0.00%
CAD, *n* (%)	26	(8.7)	26	(8.7)	1.00	(0.57–1.77)	1.000	0.00%
CHF, *n* (%)	1	(0.3)	1	(0.3)	1.00	(0.06–16.06)	1.000	0.00%
DM, *n* (%)	71	(23.8)	71	(23.8)	1.00	(0.69–1.46)	1.000	0.00%
ESRD, *n* (%)	5	(1.7)	5	(1.7)	1.00	(0.29–3.49)	1.000	0.00%
GCS, median (IQR)	13	(8–15)	13	(7–15)	-		0.878	1.26%
ISS, median (IQR)	16	(16–25)	16	(16–24)	-		0.819	1.87%
Outcomes								
Mortality, *n* (%)	47	(15.8)	35	(11.7)	1.41	(0.88–2.25)	0.154	-
Hospital stay (days)	19.5	±16.7	17.4	±16.5	-		0.121	-

CAD = coronary artery disease; CHF = congestive heart failure; CI = confidence interval; CVA = cerebral vascular accident; CRP = C-reactive protein; CLR = CRP (mg/L)/ lymphocyte (10^9^/L); DM = diabetes mellitus; ESRD = end-stage renal disease; GCS = Glasgow Coma Scale; HTN = hypertension; IQR = interquartile range; ISS = injury severity score; OR = odds ratio; SD = standardized difference.

## Data Availability

The de-identified data can be provided for academic research purposes via the corresponding author.
